# Mature Cystic Teratoma: An Integrated Review

**DOI:** 10.3390/ijms24076141

**Published:** 2023-03-24

**Authors:** Luping Cong, Sijia Wang, Suet Ying Yeung, Jacqueline Ho Sze Lee, Jacqueline Pui Wah Chung, David Yiu Leung Chan

**Affiliations:** 1 Assisted Reproductive Technology Unit, Department of Obstetrics and Gynecology, Faculty of Medicine, The Chinese University of Hong Kong, Hong Kong SAR 999077, China; lupingcong@link.cuhk.edu.hk (L.C.);; 2 Department of Obstetrics and Gynecology, Faculty of Medicine, The Chinese University of Hong Kong, Hong Kong SAR 999077, China

**Keywords:** mature cystic teratomas, benign ovarian tumors, germ cell tumors, malignant, target cancer therapy

## Abstract

Ovarian dermoid cysts, also called mature cystic teratomas (MCTs), account for 69% of ovarian germ cell tumors in young women. The tumors are formed by tissues derived from three germ layers, and sebaceous materials are most commonly seen. The origin of MCTs is widely considered to be the germ cell origin, which completes meiosis I. The clinical symptoms vary widely, but 20% of tumors could be asymptomatic. The diagnosis of MCTs is usually made without difficulty by ultrasound and confirmed by histopathology post-operatively. The imaging findings have a high diagnostic value. The typical characteristics present in the sonographic images, including a dermoid plug or Rokitansky nodule, are considered strong evidence for a teratoma. Although the malignant transformation of MCTs is rare, it can occur in some cases, especially in women of advanced age. The treatment of MCTs depends on the risk of malignancy, the age of the patient, and the patient’s fertility reserve requirement. In this article, we review the epidemiology, clinical symptoms, diagnosis criteria, cellular origin, and treatment of mature cystic teratomas.

## 1. Introduction

Ovarian dermoid cysts, which are also called mature cystic teratomas (MCTs), are the most common ovarian germ cell tumors in young women [1]. They can occur in women of different ages, while the highest prevalence was registered among women who are at their reproductive age [1]. The patients can be asymptomatic or present with chronic pelvic pain or a pelvic mass. Acute pain occurs when there is a complication. Approximately 20% of patients are asymptomatic when the tumor is discovered. The tumors are usually found incidentally on imaging performed for other indications [2,3].

The tumors are formed by tissues derived from completely differentiated cells from three germ layers, which are ectodermal, mesodermal, and endodermal [4]. Ectodermal tissues and sebaceous material are commonly encountered and present in almost every case. 38% of tumors only contain skin and neural tissues, 30% exclusively have skin and dermal appendages, and the rest have other fully differentiated histologic tissues. A cystic echo with an echogenic nodule characterized by echogenic sebaceous material and calcifications is the most common ultrasound appearance of a mature teratoma [5,6].

The possible cause of this tumor is still unclear. The reported risk factors include late menarche and long duration of menstrual irregularities, a history of cystic teratoma, fewer pregnancies, infertility, excessive drinking, and exercise [7]. Recent studies have reported that the cellular origins of mature teratomas are diploid, which contain sets of homozygous alleles. The analysis of polymorphic markers showed a homozygous pattern in teratoma components, supporting a parthenogenetic origin from post-meiotic germ cells [8]. Karyotypic analysis frequently showed a normal karyotype, 46, XX. However, tetraploidy and structural rearrangement have also been reported [1].

The management of a mature teratoma depends on the symptoms, imaging results, and the patient’s wishes. Laparoscopy and laparotomy would be used in different cases after comparing the benefits and risks [9]. Typically, cystectomy by laparoscopy is a safe and effective option to preserve ovarian function [10]. Laparotomy and oophorectomy should be considered in women with a high malignant risk [3]. Post-operative follow-up is essential to exclude recurrence, adhesion, and malignant transformation [9]. Controversy exists about the optimum surgical procedure for the treatment of ovarian dermoid cysts, especially with small cysts. Some suggest that it is practical to conduct close follow-up instead of intervention in children and adolescents with small-size mature cystic teratomas [11].

Malignant transformation of mature cystic teratoma is a rare complication with an incidence of 1–2% and is specifically seen among postmenopausal women [12]. Squamous cell carcinoma is the most common, accounting for approximately 80–85% of malignant transformations [13].

This article will review the epidemiology, clinical presentation, imaging features, diagnosis criteria, and management of mature cystic teratomas in considerable detail and aim to provide a comprehensive understanding of one of the most common neoplasms in women before menopause.

## 2. Epidemiology

There are three types of ovarian teratomas: mature teratomas, immature teratomas, and monodermal teratomas [14]. Mature teratoma includes mature cystic teratoma, which is also named ovarian dermoid cysts [15]. The reported incidence is about 1.2–14.2 cases per 100,000 people per year [16]. Mature teratoma is the most common subtype of ovarian germ cell tumors, which accounts for 11% of all ovarian tumors, 69% of all germ cell tumors, and 95% of teratomas [1,17]. The predominant type of mature teratoma is cystic, and the cyst always contains skin, hair, neural tissues, and sebaceous material. Therefore, it is also called a mature cystic teratoma (MCT) or an ovarian dermoid cyst. Mature teratomas are frequently diagnosed in the reproductive years. The patients’ ages range from 13 to 76, with a median age of mid-30 [2]. Some of them are detected during a routine examination or found during pregnancy. Although the proportion of malignant germ cell tumors in children and adolescents is larger than in adults, mature teratoma is still the predominant one, consisting consists of 61% gonadal germ cell tumors (GCTs) when compared with germ cell malignancy [18,19]. Immature teratomas, which account for over 20% of malignant germ cell tumors, also commonly arise in children and women of reproductive age and become progressively less common in postmenopausal women [20]. The right-sided tumors outnumbered the left-sided tumors [2]. The reported rate for bilateral dermoid cysts is 8–15% [3].

Some studies focus on the risk factors of benign ovarian teratomas and explore whether they share the same risk factors as testicular germ cell tumors. Unexpectedly, an increased risk of benign teratoma is associated with alcohol consumption and exercise rather than exogenous hormone exposure, which indicates that further information needs to be analyzed [7].

Although most mature cystic teratomas are benign, the reported incidence of malignant transformation is 0.5% to 3% within the MCT, especially in women of advanced age [12,13]. Squamous cell carcinoma is the most common type [16,21]. Other less frequent malignant transformations include mucinous carcinoma [21], adenocarcinoma [22,23,24,25,26], melanoma [27], carcinoid [28,29,30], oligodendroglioma [31,32,33], and sarcoma [34,35,36,37].

## 3. Clinical Symptoms

The clinical presentation of tumors is variable. About 20% of mature teratomas are asymptomatic at the time of diagnosis and are usually detected incidentally during imaging examinations, pregnancy, or abdominal or pelvic surgery for other reasons [2,3]. Larger tumors may present with abdominal pain, symptoms of increased pelvic pressure, and a palpable mass during abdominal examination [3]. Acute abdominal pain accounts for 5–10% of all matured teratomas and is frequently caused by ovarian torsion. Patients may also experience nausea, vomiting, fever, and abnormal bleeding [3]. The spontaneous rupture rate of these tumors was reported to be 1.2% to 3.8% which may lead to chemical peritonitis [38,39]. There are several risk factors for spontaneous dermoid rupture, including prolonged pressure during pregnancy, cyst torsion with infarction, or direct trauma [38,40].

Additionally, some patients with mature teratoma may present with N-methyl-d-aspartate (NMDA) encephalitis, which is a rarely seen but severe neurological disorder [41]. Several studies showed that young women or children with ovarian teratoma could be affected by encephalitis, which leads to a wide range of neuropsychiatric symptoms such as psychosis, memory loss, and behavior disorder and subsequently develops into seizures, dyskinesias, and autonomic instability [42,43,44,45,46,47]. The majority of the teratomas are mature, while the other third are immature. It has been reported that Paraneoplastic Neurological Syndrome (PNS) often precedes the clinical manifestation of ovarian teratomas [48]. The pathogenesis underlying this process might be the presence of nerve tissue expressing the NR2 subunit of the NMDA receptor in all teratomas [43]. It is likely that the cross-presentation of the same antigen explains the relationship between ovarian teratomas and anti-NMDAR encephalitis.

The incidence of pregnancy occurring in benign cystic teratoma is about 10.5%, but no evidence demonstrates that pregnancy will increase the incidence of infection, rupture, or malignancy of this ovarian neoplasm [38]. Mature ovarian cystic teratomas have rarely been reported to secrete HCG [49]. A case report found a 41-year-old woman with a known cystic lesion on her right ovary who had an elevated serum level of human chorionic gonadotrophin (hCG), and the level of hCG resolved after cyst removal [50]. Cases have been reported of a mixed polyembryoma and immature teratoma resulting in elevated serum hCG and alpha-fetoprotein [51]. Therefore, if an elevated serum hCG is found in the presence of mature ovarian teratomas, pregnancy and other malignancies such as dysgerminomas, polyembryomas, placental site trophoblastic tumors, or choriocarcinomas should be excluded.

About 15% of MCTs incidentally found thyroid tissue within the teratomas [21], but a struma ovary is termed when thyroid tissue is the predominant element (>50%) [52]. Teratomas with thyroid tissues may present with symptoms of hyperthyroidism [53].

## 4. Diagnosis

Ovarian teratomas are often diagnosed during routine gynecologic examinations. Mature teratomas can be easily diagnosed by ultrasound, as the stereotypical sonographic features are uncommonly seen in malignancies. A computed tomography scan (CT scan) and a magnetic resonance imaging (MRI) scan can sometimes also help make a diagnosis. However, it is essential to recognize the presence of any malignant changes, as their identification is of paramount importance for management and prognosis.

### 4.1. Image Findings

Ultrasound (US) is the most commonly used imaging method to confirm ovarian dermoid cysts and is accurate enough to make a diagnosis that can be confirmed by post-operative histopathology [54]. A dermoid plug or Rokitansky nodule is considered strong evidence for a teratoma [14]. It is characterized by one or more highly echogenic nodules within the cyst [14]. Plug size varies from one to almost accounting for the entire cyst [55]. A discrete, highly echogenic focus with posterior shadowing indicating the existence of the ectopic tooth might be more specific for the diagnosis of MCTs [14]. Some other sonographic characteristics include the dermoid mesh, tips of iceberg signs, and fat-fluid level [50]. CT and MRI are alternative methods of diagnosing dermoid cysts, both of which are more sensitive to fat than ultrasound [50]. The typical image findings are summarized in Table 1.

### 4.2. Histological Examination

**Figure 1:**
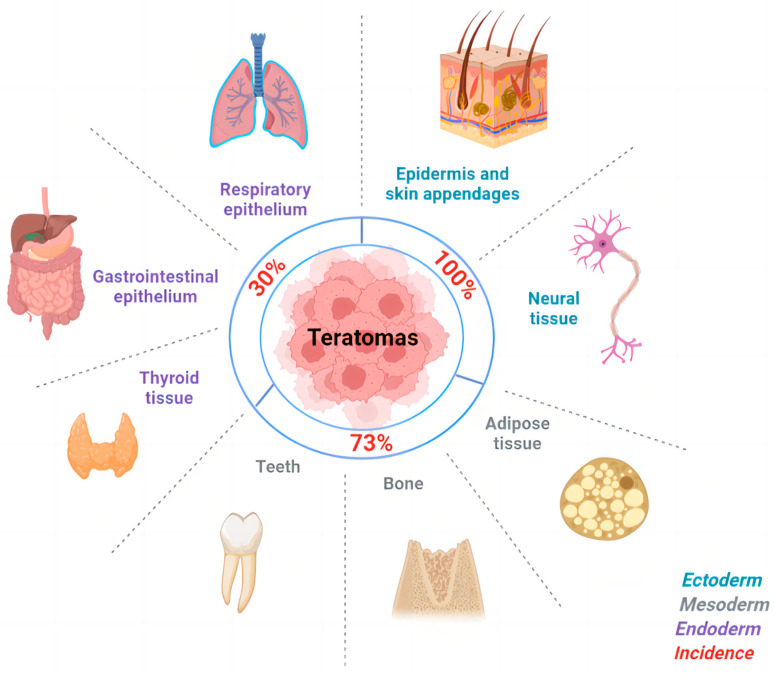
The incidence of three germ layers and the tissues in MCTs.

The gross, pathologic appearance of mature cystic teratomas is typical. The size of tumors varies, with a mean diameter of 7 cm, and they frequently contain well-differentiated cells from three germ layers [56]. The incidence of the three germ layers and the tissues that are commonly seen are summarized in Figure 1. Histologically, squamous epithelium lines the wall of the cyst, and the external surface is covered by the ovarian stroma [57]. In 88% of cases, tumors are unilocular with Rokitansky nodules, which contain hair, teeth, and other tissues [58,59]. The tumor cyst is filled with sebaceous material, which is liquid at body temperature and semisolid at room temperature [38]. Ectodermal tissue could be found in almost 100% of cases, such as the skin, but neural tissue is present in less than half of cases [2,38,57,58]. Fat, bone, cartilage, and muscle as mesodermal tissue are represented in over 90% of cases. Endodermal tissue is less seen compared with the previous two [38]. The cavity usually contains sebum, keratin, and floating hair [38]. 67–75% of cases have adipose tissue, and 31% of cases contain teeth [38,59].

### 4.3. Differential Diagnosis

Ultrasonography can be used to diagnose most mature ovarian teratomas, which are characterized by echogenic sebaceous material and calcifications [15]. However, the sonographic appearances of immature teratomas and malignant transformation are nonspecific and not enough to make a differential diagnosis. In general, these malignancies have a nonspecific clinical presentation and vary with the tumor stage [60]. Histologically, immature teratomas are usually larger, with a mean diameter of 18 cm [3]. They might be completely solid or with a few cystic components [61]. In MCTs with malignant transformation, there are septums and capsules on the surface of cysts instead of a smooth surface. The transmural growth of the Rokitansky nodule may indicate malignant changes [6]. Microscopically, immature teratomas may contain tissues from all three germ layers, but neuroepithelium is more commonly seen within the tumors when compared with their benign counterparts, and necrosis is suspected to be a sign of malignancy [56]. For the imaging findings, adhesion to neighboring structures increased wall thickness, and the presence of necrosis and hemorrhage might be a sign of malignant transformation. The tumor markers such as squamous cell carcinoma (SCC) antigen, CA 125, CA19-9, CEA, and AFP may be elevated when the malignancy exists [13,26,27,62].

## 5. Cellular Origin

The tissues within the tumor of MCTs, differentiated from cells from three germ layers, have attracted the interest of many researchers. Grossly, MCTs usually have one or more cysts containing sebaceous material, hair, teeth, and sometimes neural tissues [2,38,56]. Early cytogenetic analysis revealed that, although usually the karyotype of these cysts was 46, XX, they frequently differed genetically from their host [1,63], and trisomy, triploidy, and mosaicism have also been seen in some cases [64,65].

Centromere markers and enzyme analysis are the commonly used methods to study the karyotype of the tumor tissues from MCTs. Several studies have demonstrated that cells extracted from teratomas were frequently homozygous for enzyme makers who were heterozygous in host cells [64,66,67,68]. Moreover, the results of centromere marker analysis also resemble the enzyme analysis findings, which showed homozygous in the teratomas but heterozygous in host cells [64].

In 1975, Linder et al. investigated the origin of mature cystic teratomas from six cases using electrophoretic enzyme polymorphisms combined with centromeric chromosome heteromorphisms. The centromere was shown to be homozygous when it was heterozygous in host cells in all cases [67]. In 1978, Patil and colleagues studied 21 cases of benign ovarian teratomas. The centromeric markers in every teratoma were homozygous, while the distal markers were heterozygous or homozygous [69]. In 1982, Carritt et al. reported a case of multiple cysts in one patient. They found that four of the tumors had centromeric markers that were the same as those found in the host. Their results exclude the suppression of meiosis II as a mode of origin for some of these tumors, and they hypothesized that failure of meiosis I could also be the mechanism of tumorigenesis [70]. A study by Parrington et al. (1984) found that 13 out of 21 benign ovarian teratomas had homozygous centromeric markers and eight had heterozygous centromeric markers where the host was heterozygous. However, 11 of the 13 teratomas had both homozygous chromosomal and enzyme markers. Parrington et al. proposed that it might be due to the endoreduplication of a mature ovum [71]. In 1987, a study conducted by Surti U reported the first cases of tetraploidy and structural rearrangement in benign ovarian teratomas. They divided the origin of teratomas into four types, including failure of meiosis I (type 1) and meiosis II (type 2), endoreduplication of a mature ovum (type 3), and mitotic division of primitive germ cells (type 4) [72]. As many as 65.2% of the benign teratomas showed homozygous centromeric markers [1]. Later in 1999, Vortmeyer AO et al. conducted a tissue microdissection followed by genetic analysis of mature cystic teratomas of the ovary, and their results strongly supported Linder’s original hypothesis of a germ cell origin, which completes meiosis I [68]. Recently, a study conducted by Wang WC and Lai YC (2016) [73] proposed that mature teratomas in Taiwan are derived from premeiotic ovarian germ cells [73]. However, based on the evidence that both components show a homozygous pattern by molecular analysis, which strongly demonstrated a post-meiosis I oocytes origin, the reason for the discrepancy might be the host somatic cell contamination [8,74]. In summary, the studies of the origin of MCTs are listed in Table 2 chronologically.

## 6. Treatment

The management of a mature teratoma is influenced by the risk of malignancy, the age of the patient, and the fertility reserve requirement. Surgical removal is an effective treatment for ovarian dermoid cysts. Chemotherapy and targeted drug therapy are considered when there is any malignant change or when it is combined with other ovarian cancers.

### 6.1. Mature Cystic Teratomas

Surgical removal is an effective treatment for ovarian dermoid cysts. The surgeries were classified as ovary-sparing surgery (OSS) or oophorectomy, either laparoscopic or via laparotomy [75]. Recently, the increasing use of laparoscopy in gynecologic surgery required a reexamination of the management of MCTs [9]. Compared with traditional laparotomy, the potential benefits of laparoscopy include a reduction in post-operative pain, blood loss, and a shorter hospital stay [76]. However, laparoscopy has been criticized for being associated with a higher risk of intraperitoneal cyst rupture that increases the risk of chemical peritonitis and adhesion formation, and an erroneous diagnosis may cause the iatrogenic spill of malignant cells [77,78]. The published rupture and spillage rates with laparoscopic surgery vary widely, from 0% to 100% [10].

Generally, cystectomies are performed only to remove the cysts so that they can preserve the rest of the ovarian function; however, follow-up is necessary to exclude recurrence, which occurs in 4% to 5% of cases [9]. It is considered that teratomas with a diameter of around 5 cm in premenopausal women require surgical resection, but a smaller tumor will also warrant removal in the postmenopausal woman because of the higher risk of malignant transformation [79]. Oophorectomy is the standard treatment in postmenopausal women and should be considered in perimenopausal women with multiple teratomas in one ovary and women with a large cystic teratoma destroying large normal ovarian tissue [80]. Since MCTs are the most common ovarian germ cell tumors in children and adolescents, it is important to make the best compromise between the preservation of fertility and the prevention of recurrences [75]. In children, the low malignancy rate leads to increased consideration of ovarian sparing surgery (OSS) as a better option for MCTs [81]. OSS should always be considered in the following cases: no evidence of lymphadenopathy or liver/lung metastasis, normal levels of tumor markers, absence of calcifications, or specific radiological findings [75]. Ovarian masses in postmenopausal women, even those with imaging findings of MCT, should be carefully evaluated if they are accompanied by any malignant changes because of the early stage of the tumor. No clinical, radiological, or biological sign is specific. Therefore, resection of any ovarian mass, even if asymptomatic, is required [13,82].

### 6.2. Malignant Transformations

**Figure 2:**
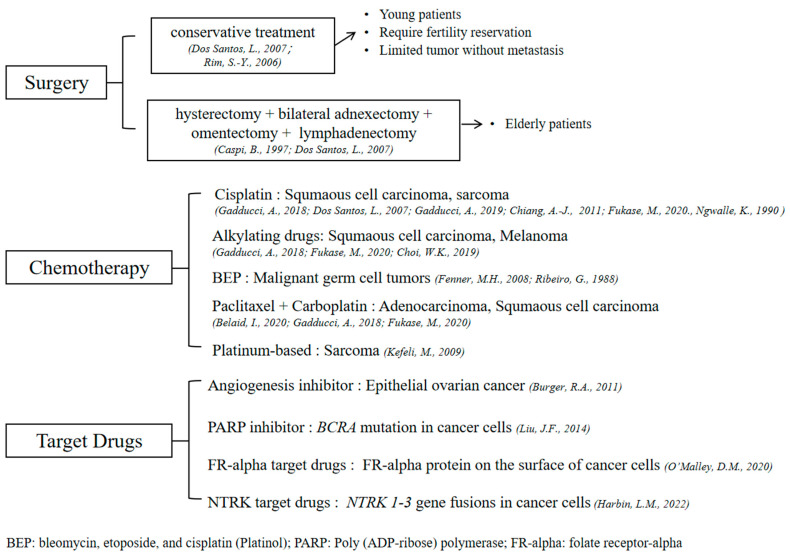
Treatment options for malignant transformations [3,21,22,27,34,35,79,85,86,87,88,89,90,91,92,93,94].

Mature cystic teratoma is the most common type of ovarian germ cell neoplasm, but occasionally it can undergo malignant transformations, especially in postmenopausal women over 40 years old [82,83]. Although imaging examinations can diagnose MCTs with considerable accuracy, tissue examination is necessary to exclude any malignancy [11]. The majority of malignant transformations are squamous-cell carcinomas, while the remainders are carcinoid tumors, adenocarcinomas, melanoma [27], oligodendroglioma [31,32,33], and sarcoma [16,34,35,36,37,83,84,85]. The treatments for these malignant transformations are the same as for ovarian carcinoma, including surgical removal and chemotherapy. Radiotherapy did not show benefits for prognosis [16,21,84]. The treatment options for malignant transformations are summarized in Figure 2.

For young patients who wish to maintain their fertility, the malignant transformed dermoid cysts limited to the ovarian capsule without local or distant invasion can be treated by conservative treatment by conducting unilateral adnexectomy with multiple peritoneal biopsies [85,86]. In cases of elderly patients, the treatment is the same for any ovarian cancer, including total hysterectomy with bilateral adnexectomy, omentectomy, and lymphadenectomy [79,86]. The best adjuvant therapy for squamous cell carcinomas derived from MCTs has not been determined, but cisplatin is commonly used [84,86,87,88]. Alkylating drugs were also found to be associated with significant increases in survival in tumors at stages beyond Ia in Hackethal’s study in 2008 [16]. Paclitaxel and carboplatin are used in patients with adenocarcinoma [22]. Dacarbazine (DTIC) monotherapy or combination therapy, including nitrosourea and cisplatin, may be administered as adjuvant therapies for patients with melanoma [27]. Cisplatin and adriamycin were used in patients with sarcoma [34]. Patients with other ovarian germ cell tumors can achieve complete remission after BEP treatment, which is the combination of bleomycin, etoposide, and cisplatin (Platinol) [89,90].

Target cancer therapy as precision medicine is to target specific genes and proteins in tumor cells to stop their growth and survival. For germ cell tumors (GCTs), this aim is challenging since it varies in histological subtypes and the low number of relapse cases [95]. However, there are several target cancer therapy drugs for ovarian cancers that could be taken into consideration when there is malignancy accompanied by MCTs. Angiogenesis inhibitors are used to prevent the growth and spread of cancers. Bevacizumab has been proven to be effective against epithelial ovarian cancer [91]. Poly(ADP)-ribose polymerase (PARP) inhibitors, such as Olaparib, Rucaparib, and Niraparib, may result in the accumulation of DNA damage and result in selective cell death, particularly if a *BRCA* mutation is detected in cancer cells [92]. Antibody-drug conjugates (ADC) are chemotherapy drugs linked with antibodies. Many epithelial ovarian cancer cells have high expression levels of the surface folate receptor-alpha (FR-alpha) protein. In this situation, ADC can attach to the FR-alpha protein and deliver chemotherapy drugs to cancer cells [93]. Changes in neurotrophic tyrosine receptor kinases (*NTRK*) genes, specifically the *NTRK 1-3* gene fusions, appear in a few ovarian cancers. Targeted drugs such as Larotrectinib and Entrectinib can combine with the NTRK receptor on the cell surface and subsequently inhibit the activation of downstream pathways, which can prevent cancer cell proliferation and induce apoptosis [94]. However, in some refractory tumors, drug resistance is one of the main reasons for tumor relapse and metastasis. The mechanisms of drug resistance are various, mainly including muli-drug resistance, apoptosis suppression, altering drug metabolism, epigenetic and drug targets, enhancing DNA repair, and gene amplification [96].

## 7. Conclusions

Mature cystic teratomas are one of the most common ovarian tumors among young women. These tumors are composed of tissues that are derived from ectoderm, mesoderm, or endodermal layers. Clinical presentations are associated with tumor size and complications. Image examination is extremely helpful in diagnosis and management. The treatment for MCTs should be based on the clinical symptoms, the risk of malignancy, the patient’s age, and the requirement for fertility preservation. Better knowledge of the epidemiology, clinical, and imaging features of mature cystic teratomas can be helpful in making an accurate diagnosis and subsequently guiding clinicians to make the most appropriate treatment plan. Meanwhile, the next generation sequencing brought light to explore new drugs for those refractory tumors. However, the biggest challenge is the low malignant transformation rate in MCTs and the high cure rate after surgery. Therefore, international cooperation is necessary for basic research and clinical trials.

## Figures and Tables

**Table 1:** Typical image findings of MCTs [3,6,14,15,55].

	Pathologic Equivalent	Image Findings
US	Rokitansky nodule	Echogenic tubercle with cystic echo
	Hair and watery fluid	Thin band-like echoes
	Presence of sebum	Dense echo pattern
	Sebum layered on serous fluid	Fat–fluid level
	Desquamative material	Floating intracystic spheres
CT	Fat intralesional	Substance’s density is −20 HU or lower inside a cyst
	Rokitansky nodule	Rounded (or bridge) structure, clearly linked to the cyst wall, projecting inside the cyst
		Or
		Irregular mural thickening containing dense structures (calcified structures) and\or areas of fatty
MRI	Sebaceous material	T1 hyperintense and T2 hypointense
	Squamous material	T1 hypointense and T2 hyperintense
	Hair	T2 hypointense
	Tooth	Low-signal-intensity central calcifications

US: Ultrasound; CT: Computed tomography; MRI: Magnetic resonance imaging.

**Table 2:** The studies of the cellular origin of MCTs.

References	Materials	Markers	Main Results	Conclusion
Linder D et al. [66] 1970	39 MCTs from 33 patients	Allelic isozymes: G6PD, PGM1, PGM3, 6PGD	6 of 12 tumors at PGM1 locus were heterozygous in host but homozygous in tumors	MCTs are from post-meiotic origin
Linder D et al. [67] 1975	5 MCTs from 5 patients	Q-banding, G-banding, C-banding; PGM1, PGM3, 6PGD, etc.	2 of 3 tumors at PGM1 locus, and 1 of 2 tumors at PGM3 locus were heterozygous in host but homozygous in tumors	MCTs arise from single germ cell after first meiosis
Eppig JJ et al. [64] 1977	23 teratomas from strain LT/SV mice	Gpi-1	21 of 23 tumors at Gpi-1 locus were heterozygous in host but homozygous in tumors	Tumors are origin from post-meiotic oocytes
Carritt B et al. [70] 1982	7 benign ovarian teratomas in one patient	C-banding, PGM1, PGD, UMPK, ME2	4 of 7 teratomas showed heterozygous on c-banding results	Exclude the suppression of meiosis II as amode of origin for some of these tumors.
Parrington JM et al. [71] 1984	21 benign ovarian teratomas from 14 patients	C-banding, Q-banding; 13 enzyme markers (PGD, PGM1, PGM3, etc.)	52% of teratomas had homozygous centromeres and enzymes	Many of this group are thought to arise from duplication of a mature ovum
Surti U [1] 1990	102 benign mature teratomas from patients	Q-banding, C-banding	First reported tetraploidy and structural rearrangement in teratomas; 65% of teratomas have homozygous centromere	65% of tumors arose from single germ cell after first meiosis or failure of second meiosis or endoreduplication of mature ovum; the rest arose by failure of first meiosis or mitotic division of premeiotic germ cell
Vootmeyer AO [68] 1999	7 mature ovarian teratomas	Microdissection; PCR using microsatellite markers	Markers showed homozygous in majority tumors	Tumors arose from post-meiotic germ cell
Wang WC [73] 2016	9 MCTs	15 STR analysis; Methylation analysis	Most of the STR loci in tumors showed heterozygous	Exclude the parthenogenetic origin of MCTs

MCTs: Mature cystic teratomas; G6PD: glucose-6-phosphate dehydrogenase; PGM1: Phosphoglucomutase 1; PGM3: Phosphoglucomutase 3; 6PGD: 6-phosphogluconate dehydrogenase; Gpi-1: Glucose-6-phosphate isomerase 1; UMPK: Uridine monophosphate kinase; PCR: Polymerase chain reaction; STR: Short tandem repeat.
